# Deep and dynamic metabolic and structural imaging in living tissues

**DOI:** 10.1126/sciadv.adp2438

**Published:** 2024-12-11

**Authors:** Kunzan Liu, Honghao Cao, Kasey Shashaty, Li-Yu Yu, Sarah Spitz, Francesca Michela Pramotton, Zhengpeng Wan, Ellen L. Kan, Erin N. Tevonian, Manuel Levy, Eva Lendaro, Roger D. Kamm, Linda G. Griffith, Fan Wang, Tong Qiu, Sixian You

**Affiliations:** ^1^ Research Laboratory of Electronics, MIT, Cambridge, MA 02139, USA.; ^2^ Department of Electrical Engineering and Computer Science, MIT, Cambridge, MA 02139, USA.; ^3^ Department of Biological Engineering, MIT, Cambridge, MA 02139, USA.; ^4^ Department of Brain and Cognitive Sciences, MIT, Cambridge, MA 02139, USA.; ^5^ Department of Mechanical Engineering, MIT, Cambridge, MA 02139, USA.

## Abstract

Label-free imaging through two-photon autofluorescence of NAD(P)H allows for nondestructive, high-resolution visualization of cellular activities in living systems. However, its application to thick tissues has been restricted by its limited penetration depth within 300 μm, largely due to light scattering. Here, we demonstrate that the imaging depth for NAD(P)H can be extended to more than 700 μm in living engineered human multicellular microtissues by adopting multimode fiber-based, low repetition rate, high peak power, three-photon excitation of NAD(P)H at 1100 nm. This is achieved by having more than 0.5 megawatts peak power at the band of 1100 ± 25 nm through adaptively modulating multimodal nonlinear pulse propagation with a compact fiber shaper. Moreover, the eightfold increase in pulse energy enables faster imaging of monocyte behaviors in the living multicellular models. These results represent a substantial advance for deep and dynamic imaging of intact living biosystems. The modular design is anticipated to allow wide adoption for demanding imaging applications, including cancer research, immune responses, and tissue engineering.

## Teaser

Accessible multimode fiber sources enable 720-μm deep NAD(P)H imaging of intact living human multicellular microtissues.

## INTRODUCTION

Capturing the metabolic dynamics of intact and living biosystems is essential in biomedicine from fundamental research to clinical pathology. Label-free, two-photon autofluorescence (2PAF) imaging of reduced form nicotinamide adenine dinucleotide phosphate [NAD(P)H] and flavin adenine dinucleotide (FAD) enables nondestructive, high-resolution, and three-dimensional (3D) visualization and characterization of cellular metabolic activities. The use of two-photon (2P) excitation of NAD(P)H imaging has been adopted in major tissue types and diseases for noninvasive assessment of oxidative phosphorylation and glucose catabolism in living cells (*1*–*8*). However, NAD(P)H imaging rarely extends beyond 300 μm due to light scattering and out-of-focus background (table S1) (*2*, *4*, *9*). Compared to the widely adopted 2P-based labeled imaging (*10*), this problem of limited depth penetration in NAD(P)H imaging is exacerbated by two intrinsic properties of NAD(P)H autofluorophore: (i) 2P cross section of NAD(P)H is approximately three to four orders of magnitude lower than the commonly used labels such as fluorescent proteins [10^−2^ Goeppert Mayer (GM) of NAD(P)H (*11*) compared to 10^1^ to 10^2^ GM of calcium green-1, calcium crimson, and calcium orange with Ca^2+^ (*12*)]; (ii) the molecular structure and energy levels (ground electronic state and excited electronic states) of NAD(P)H require more blue-shifted excitation wavelengths compared to existing markers [~750 nm for NAD(P)H (*11*) compared to ∼930 nm for green fluorescent proteins (*12*)], leading to increased scattering in deep tissue.

Three-photon (3P) excitation of fluorescent markers in label-based imaging has been proposed and demonstrated to substantially reduce scattering and enhance the signal-to-background ratio (SBR) in deeper tissues (*13*–*18*). Nevertheless, this deep imaging has not been successfully extended to label-free NAD(P)H imaging. Prior work, abbreviated as SLAM (simultaneous label-free autofluorescence-multiharmonic) imaging, demonstrated the possibility of 3P excitation of NAD(P)H using photonic crystal fiber (PCF)–based 1110-nm sources (*9*). The introduction of 1110-nm excitation enabled simultaneous acquisition of third harmonic generation (THG) signals from structural interfaces, such as water-lipid interface (e.g., intracellular and extracellular membranes) and water-protein interface (e.g., extracellular matrix and nucleoli) (*19*–*21*), together with the 3P excitation of NAD(P)H and 2P excitation of FAD, plus second harmonic generation (SHG) from collagen fibers (*22*, *23*). The combination of these orthogonal contrasts allowed simultaneous visualization of diverse cellular and extracellular components in the unperturbed tissue microenvironment (*24*–*28*). Nevertheless, because of the energy constraint of excitation pulses out of the PCF followed by a pulse shaper, the depth in (*9*) was limited to 200 μm.

In this work, we demonstrate for the first time that the depth limit of NAD(P)H imaging can be extended to more than 700 μm using living engineered human multicellular microtissues as test samples and by adopting multimode fiber (MMF)–based low repetition rate high peak power 3P excitation of NAD(P)H at 1100 nm, enabling deep and dynamic SLAM (dSLAM) imaging. The high peak power exceeding 0.5 MW at the band of 1100 ± 25 nm was obtained by adaptively modulating multimodal nonlinear pulse propagation with a compact fiber shaper (*29*). Furthermore, the eightfold increase of pulse energy at 1100 nm allows us to capture faster monocyte behaviors in the engineered human multicellular microtissues in vitro. These results and findings represent an important advance toward deeper and faster metabolic and structural imaging of intact living biosystems. We anticipate that the flexibility provided by the modular design [step-index (SI) MMF with a slip-on fiber shaper] will allow the proposed imaging methodology to be widely adopted for demanding in vivo and in vitro imaging applications (*30*), including cancer research (*4*, *31*), immune responses (*8*, *32*), and tissue engineering (*33*, *34*).

## RESULTS

### SI MMF for high-quality high peak power metabolic and structural imaging

**Fig. 1.**
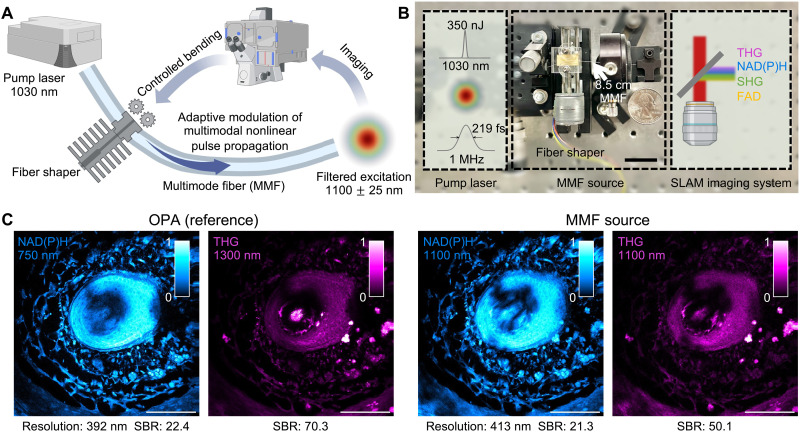
SI MMF as a compact, accessible, and high-quality label-free imaging source. (**A**) Schematic for working principles. High-energy ultrashort pulses from a pump laser (up to 350 nJ, 219 fs) at 1030 nm were injected into the SI MMF, where the propagation of multimodal nonlinear pulses was adaptively modulated by a compact fiber shaper that applied controlled macro-bending. The spectrally filtered fiber output at 1100 nm was directed to the microscopy system for label-free metabolic and structural imaging, where the imaging signal was used as the feedback for controlling the fiber shaper. (**B**) Photograph (middle) of the MMF source based on a compact slip-on fiber shaper and its comparison with a US quarter, highlighting the compactness and the modular nature of the MMF source. Left and right panels: Illustrative diagrams of the input pulses and the imaging system. Scale bar, 30 mm. (**C**) Comparison between images acquired with a commercial optical parametric amplifier (OPA) and with the MMF source at the same site of the mouse whisker pad tissue. The OPA images were captured sequentially at 750 and 1300 nm, whereas the MMF source images were captured simultaneously at 1100 nm (color bar for normalized signal intensity). Scale bars, 100 μm.

To create a high peak power and accessible fiber source at the band of 1100 nm, we chose (i) a standard silica-core SI MMF as the medium for its relatively large mode area and power scalability (*35*–*42*) and (ii) a slip-on compact fiber shaper as the control device for its low cost and ease of use (*29*) (Fig. 1, A and B).

Ultrashort pulses (Light Conversion, Carbide) at 1030 nm of 1-MHz repetition rate were launched into a 8.5-cm-long SI MMF [25 μm in core diameter, 0.1 numerical aperture (NA)] and the pulse propagation was modulated by controlled bending through a slip-on fiber shaper (see Materials and Methods). The input peak power up to 1.60 MW (350-nJ pulse energy) is well below the critical power of silica at around 4.7 MW (*43*).

**Fig. 2.**
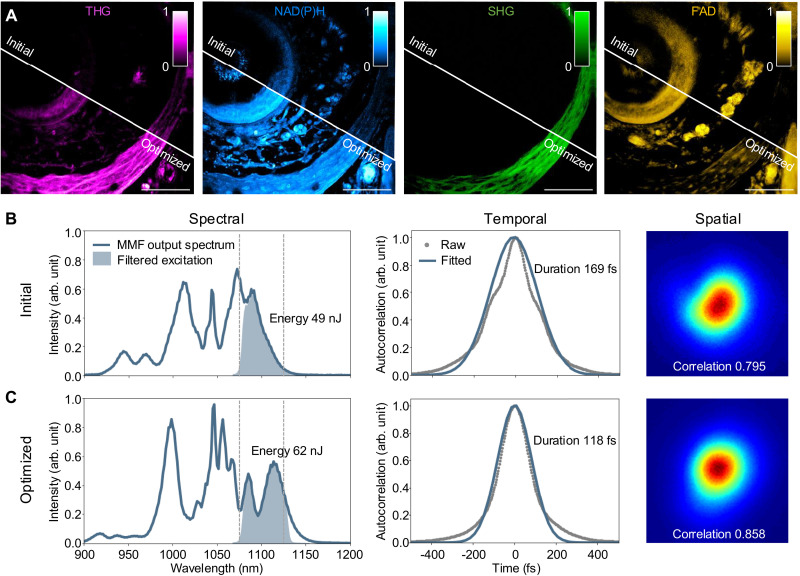
Shaping the SI MMF to optimize imaging SNR and resolution. (**A**) Comparison of images at the same site of the mouse whisker pad tissue acquired before (initial) and after (optimized) optimizing the fiber shaper, with the same contrast adjustments (colorbar for normalized signal intensity). Scale bars, 100 μm. Spectral, temporal, and spatial characterizations of the fiber output at the spectral band of 1100 ± 25 nm before (**B**) and after (**C**) optimizing the fiber shaper. The spectra before filtering are also presented (solid blue lines in the left panel). Duration is estimated based on the autocorrelation signals and with the assumption of Gaussian pulse shape. Spatial profiles represent the near-field intensity distributions of the fiber output; numbers denote the correlation with the fundamental LP_01_ mode.

As expected, the increased nonlinearity of 3P processes rendered THG and NAD(P)H imaging highly susceptible to the deteriorating beam profile, which deviated from a Gaussian beam, and to the increased temporal duration, both known characteristics of the MMF output field (*44*). Building on the recent work demonstrating the modulation of multimodal nonlinear pulse propagation (*29*), we developed a compact fiber shaper for the 8.5-cm-long, off-the-shelf SI MMF that supports fewer modes. This addresses the need for an improved beam profile (resembling a Gaussian beam) and shorter temporal duration, enabling deeper and dynamic SLAM imaging (Fig. 1B). Compared to the previous 3D printed five- to eight-actuator fiber shaper, the relatively shorter pulses are obtained by using a shorter fiber (30 cm versus 8.5 cm) and a compact laser cutting–based fiber shaper (consisting of one downsized actuator, see more in Materials and Methods). The near-Gaussian beam was obtained by modulating the multimodal pulse propagation within an MMF with smaller NA and smaller core and spectral filtering of the red-shifted wavelengths at 1100 nm. Similar to the previously demonstrated fiber shaper, changing the position of the actuator applies controlled macrobending on the fiber, which produces local refractive index perturbations and alters the propagation of the nonlinear multimodal pulses, thus customizes the MMF output in the spectral-temporal-spatial properties. By integrating the signal level of THG imaging as the real-time feedback, the actuator position can be actively adjusted to produce a spectral-temporal-spatial profile that is optimal for 3P imaging (Fig. 2).

To investigate the quality of the images that can be obtained with our MMF source, we compare the images out of the optimized MMF source with a state-of-the-art optical parametric amplifier (OPA) (Light Conversion, Cronus-3P) (NAD(P)H/THG using 750 nm/1300 nm from OPA and 1100 nm from MMF; see table S2 for acquisition parameters) at the same site of mouse whisker pad tissue (see Materials and Methods for sample preparation). The results presented in Fig. 1C show that the SBR and the resolution are comparable between the two image sets (see Materials and Methods for SBR calculation and see Materials and Methods and fig. S1 for resolution characterization). The NAD(P)H image out of the MMF source looks even more highly resolved than that of the OPA, but this is very likely to be the consequence of 3P excitation rather than a result of the difference in beam profile (see more in Result).

Because this is an end-to-end adaptive optimization, characterization of the MMF output field in the spectral, temporal, and spatial domains was performed after the imaging session to provide more information into the mechanism of the improvement. A representative illustration of this optimization process is presented in Fig. 2, where it shows that the signal enhancement of the multiphoton processes can be reliably achieved by adaptively adjusting the position of the single-actuator fiber shaper guided by the signal level of THG imaging. Specifically in this example of imaging, the mouse whisker pad tissue, from the initial to the optimized fiber shape, a 1.27-fold increase in band energy and a 1.43-fold reduction in pulse duration, with an improvement of beam spatial profile were obtained (Fig. 2, B and C, and see Materials and Methods for correlation calculation). The theoretical signal enhancement for 3P (4.16-fold) and 2P (2.29-fold), calculated using the enhancement in band energy and reduction in pulse duration (*45*), are comparable to the measured signal-to-noise ratio (SNR) enhancements from Fig. 2A, 5.22-fold in 3P processes and 2.70-fold in 2P processes (see Materials and Methods for SNR calculation). The slight discrepancy is likely due to the simple assumptions of Gaussian pulse shape and Gaussian beam shape. These results show that, by using the compact fiber shaper on the SI MMF that supports fewer modes, a 120-fs near-Gaussian fiber output can be reliably obtained for high-quality, high peak power metabolic (3PAF NAD(P)H) and structural (THG) imaging. In addition, the entire wavelength conversion unit is highly compact, modularized, and low cost (Fig. 1B). These results demonstrate the feasibility of using a standard SI MMF with a compact fiber shaper for accessible, high-quality, and high peak power excitation at 1100 nm.

### Feasibility of using 1100 nm for deep 3PAF NAD(P)H imaging

**Fig. 3.**
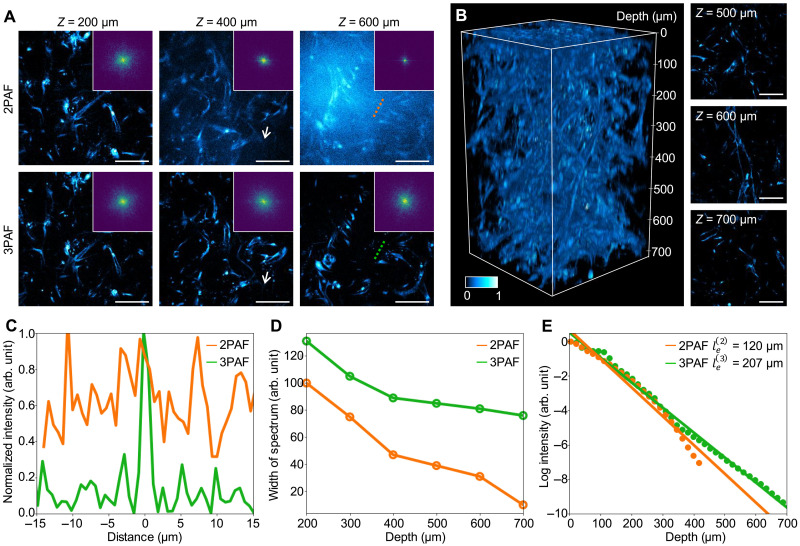
Deep NAD(P)H imaging with 1100-nm MMF source. (**A**) Comparison of 2PAF and 3PAF NAD(P)H imaging of intact 3D microvascular network at different depths, applying identical contrast adjustments for the 2PAF and 3PAF images at the same depth. Magnitude of the Fourier transform of individual images is plotted in the insets, indicating differences in the resolution of the images. (**B**) 3PAF NAD(P)H imaging of the 3D microvascular network through the entire 720 μm of depth, with 2D images shown at different imaging depths (color bar for normalized signal intensity). (**C**) Normalized intensity profiles along the coregistered lines in (A). (**D**) Width of the magnitude of the Fourier transform across different depths in 2PAF and 3PAF NAD(P)H imaging. (**E**) Signal intensity of 2PAF and 3PAF as a function of imaging depth measured in the same site of (B). The EALs $\ell_{e}^{\left(2\right)}$ and $\ell_{e}^{\left(3\right)}$ were fitted for 2PAF and 3PAF, respectively. Scale bars, 100 μm.

To investigate the depth limitation of NAD(P)H imaging and the effectiveness of using 1100 nm to extend the depth limit, we performed NAD(P)H autofluorescence imaging with 2P excitation at 750 nm and 3P excitation at 1100 nm (more characterizations of 3PAF NAD(P)H imaging can be found in fig. S2 for validation and notes S1 for estimation of 3P action cross section, and S2 for estimation of saturation pulse energy). For comparison, the 2PAF NAD(P)H imaging was performed using the signal path from the OPA at 750 nm, and the 3PAF NAD(P)H imaging at the same site was performed using the MMF-based 1100 nm source using a 3D microvascular network (*46*, *47*), with varying pulse energy according to the imaging depths (see Materials and Methods for volumetric imaging acquisition and table S2 for parameters). The microvascular network is an engineered human multicellular microtissue that consists of dense and complex 3D network formed by vascular endothelial cells, which was chosen as test samples because the vascular endothelial cells exhibited stable NAD(P)H signals through the entire 720 μm of depth (Fig. 3B).

In superficial layers (Z≤200 μm), the NAD(P)H images generated using 2PAF have comparable SNR and resolution as the 3PAF using 1100 nm. However, as the imaging delves deeper, due to the out-of-focus background signal, the SBR degrades much faster in 2PAF (Fig. 3A and see Materials and Methods for SBR calculation). For example, at the depth of Z=600 μm, the SBR of 2PAF dropped to 1.12 compared to 6.68 for 3PAF. The low SBR can lead to failure in identifying cells (white arrow in Fig. 3A at Z=400 μm) and structures (lines in Fig. 3A at Z=600 μm, with intensity profiles in Fig. 3C).

Next, we quantified the image degradation and signal attenuation of both 2P and 3P nonlinear processes in relation to imaging depth in Fig. 3 (D and E). We observed that a substantial contributor to the degradation of the 2PAF NAD(P)H imaging is the overwhelming background and the blurred high-frequency details in the spatial domain of the 2D image slice, which corresponds to a substantially increased dominance of the low-frequency zone and decreased coverage of the high-frequency zone in the frequency domain, as visualized by the spectrum of the Fourier transform shown in the insets of Fig. 3A. Qualitatively, we computed and plotted the width of the spectrum of the Fourier transform in Fig. 3D across different depths. As the imaging depth increases, the resolution degradation occurs much faster in the 750 nm 2P excitation scheme. In addition, we characterized the effective attenuation lengths (EALs) of NAD(P)H, defined by the depth at which the fluorescence signal attenuates by $1/e^{2}$ and $1/e^{3}$ for 2P and 3P imaging, respectively (*15*). The EAL of 1100-nm 3PAF NAD(P)H imaging was measured as $\ell_{e}^{\left(3\right)}=207$ μm, 1.73 times longer than that of $\ell_{e}^{\left(2\right)}=120$ μm in 750 nm 2PAF (Fig. 3E), which benefits from the use of longer excitation wavelength in 3P imaging (*13*, *14*, *16*, *48*).

These investigations suggest that NAD(P)H imaging in deep tissue requires 1100-nm high peak power 3P excitation for high-resolution and high-contrast imaging throughout the entire depth of the 3D microvascular network, underscoring the potential of dSLAM for deep and dynamic metabolic and structural imaging.

### Deep metabolic and structural imaging of living blood-brain barrier microfluidic model

**Fig. 4.**
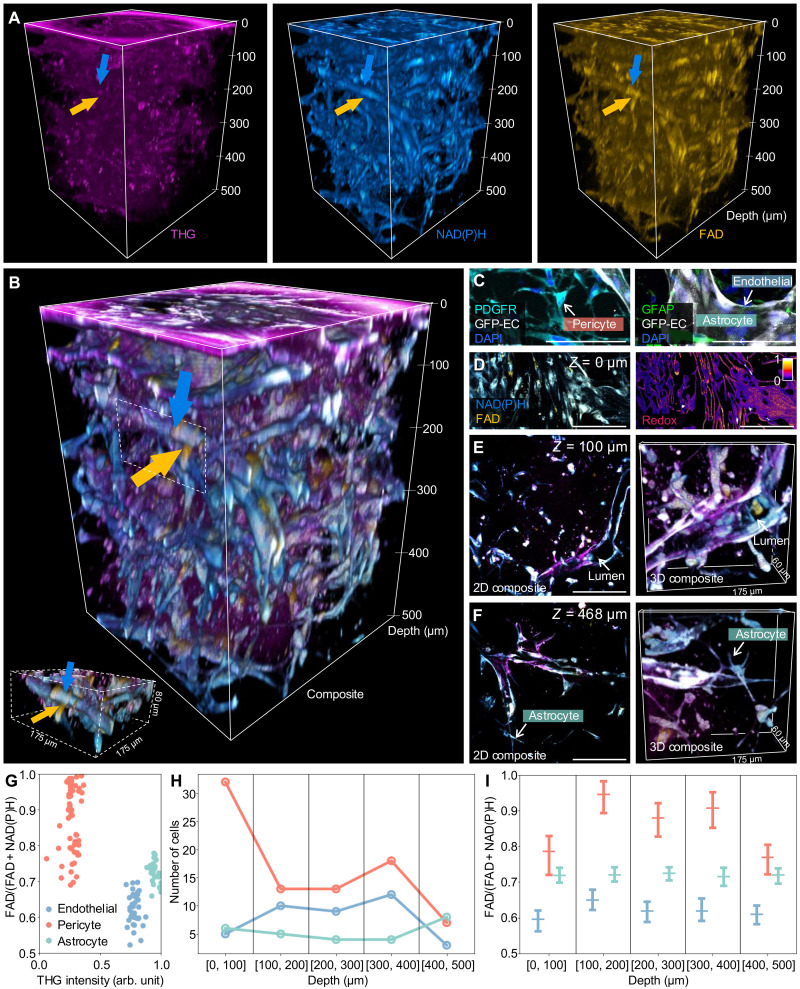
dSLAM for deep metabolic and structural imaging. 3D visualization of THG, NAD(P)H, and FAD signals (**A**) and the merged signals (**B**) for structural and metabolic imaging of the entire 500-μm-deep living blood-brain barrier microfluidic model, comprising vascular endothelial cells, pericytes, and astrocytes. Dashed volume in (B): A FAD-strong pericyte (yellow arrow) wraps an NAD(P)H-strong vascular endothelial cell (blue arrow). (**C**) Immunofluorescence (IF) imaging of stained vascular endothelial cells, pericytes, and astrocytes. (**D**) NAD(P)H and FAD imaging and the resulting map of redox ratio in subcellular resolution at the shallowest layer (color bar for redox ratio). Panels (**E**) and (**F**) are 2D and 3D visualizations at different deeper layers, showing the lumen structure and astrocytes extending projections around vascular endothelial cells in the living blood-brain barrier microfluidic model. (**G**) Redox ratio and THG intensity of cells in the volume. Cells cluster into the same type based on their structural and metabolic features. Density (**H**) and redox ratio (**I**) of different cell types at different depths of the living blood-brain barrier microfluidic model. Scale bars, 100 μm.

A major motivation for developing the dSLAM imaging platform is to perform noninvasive deep metabolic and structural characterization of living multicellular systems at the subcellular level (fig. S3). To examine the capability of dSLAM for this task, we acquired a 350 μm by 350 μm by 500 μm image stack of living blood-brain barrier microfluidic models (the entire depth of the model) (*49*), as shown in Fig. 4 (see table S2 for acquisition parameters). Developing and monitoring blood-brain barrier microfluidic models are essential for improving targeted therapies for neurological disorders (*49*). Deep label-free metabolic and structural imaging of human blood-brain barrier models in vitro could aid the development of therapeutics with improved brain delivery. Here, we show that, by using the MMF-based 1100-nm excitation with over 0.5 MW peak power, the deep NAD(P)H imaging integrated with the simultaneously acquired THG and FAD imaging allows 3D redox mapping of the entire intact living human blood-brain barrier model (Fig. 4, A and B, and see movies S1 and S2).

The human blood-brain barrier microfluidic models were developed with self-assembled vascular endothelial cells, pericytes, and astrocytes in a 500-μm-thick microfluidic device (see Materials and Methods). 3D visualization from Fiji (*50*) distinctly shows the three cell types based on their optical signatures in the surrounding fibrin gel (Fig. 4, A and B compared with immunofluorescence (IF) imaging of stained cells in Fig. 4C). For example, vascular endothelial cells, which are engineered to form a perfusable vasculature (blue arrow in Fig. 4B), exhibit high NAD(P)H, consistent with demonstrations that endothelial cells use glycolysis for adenosine 5′-triphosphate (ATP) production (*27*, *51*–*54*). Pericytes, wrapping around the vasculature (yellow arrow in Fig. 4B), are observed to have a high concentration of FAD, likely due to their contractile functions (*55*–*57*). Astrocytes, responsible for regulating ion and neurotransmitter concentrations, exhibit strong THG signals, which is in line with previous reports and might be attributed to their complex branched morphology (*52*, *58*). Further quantification of the redox ratio and the THG signal intensity in Fig. 4G shows distinct cell clusters based on their raw optical signatures.

Together with the distinct optical signatures, the deep 3D imaging capability enables noninvasive visualization of the complex multicellular interactions, metabolic states, and depth-dependent variations within the living blood-brain barrier microfluidic model (see Materials and Methods and fig. S4 for cell segmentation and classification). At the shallowest layer (Z=0 μm, closest to the cover glass bottom), the cells displayed flat-out morphology that is similar to 2D cell cultures (Fig. 4D). As the imaging delves deeper, cells start to exhibit 3D features, such as the forming of the lumen at Z=100 μm (Fig. 4E, arrow pointing to the lumen of the vasculature network formed by vascular endothelial cells), the wrapping actions of pericytes over vascular endothelial cells at Z=120 μm (Fig. 4B, dashed volume with zoom-in visualization on the side, with yellow arrow pointing to the pericytes and blue arrow pointing to the vascular endothelial cells), and astrocytes extending projections around vascular endothelial cells at Z=468 μm (Fig. 4F). Furthermore, deep structural and metabolic imaging reveals depth-dependent metabolic distributions in living cells. For example, among all the 149 cells analyzed in the imaging volume (Fig. 4I), pericytes located in the middle layers of the blood-brain barrier model (Z=100 to 400 μm) exhibited higher FAD intensity compared to those in the outer layers. This phenomenon may arise from the proximity of the glass chamber at the shallowest and deepest layers of the blood-brain barrier microfluidic model, which could affect the pericyte contractile functions, or limit the growth of pericyte due to the constrained orientation of the vasculature network.

### Dynamic metabolic and structural imaging of monocyte behaviors in vitro

Another motivation for developing a label-free metabolic and structural imaging system is to perform noninvasive imaging of cellular dynamics in living and thick biosystems. Previous SLAM imaging demonstrates the capability of SLAM microscopy on tracking the metabolic dynamics of leukocyte migration in vivo up to 40 μm/min (*9*). The speed was largely limited by the available peak power (∼40 kW) at the excitation focus (fig. S3). With the peak power of more than 0.5 MW out of filtered excitation of the optimized MMF source, we are able to record time-lapse videos at a pixel rate between 0.5 and 1 MHz, which is ultimately limited by the repetition rate of our pump laser.

**Fig. 5.**
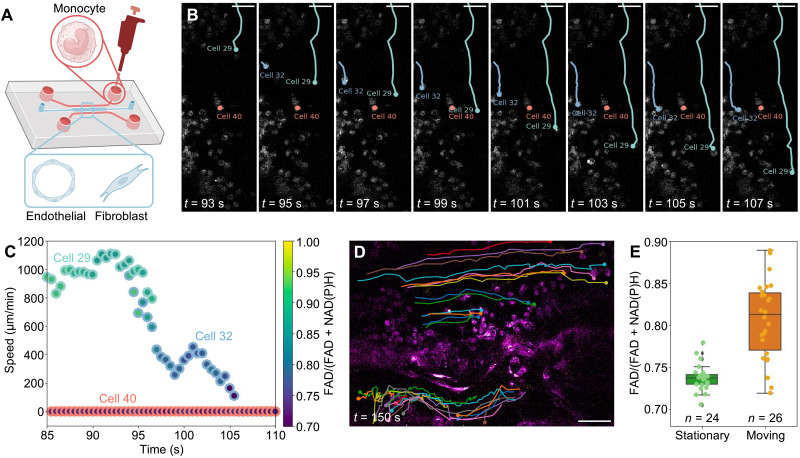
dSLAM for dynamic metabolic and structural imaging. (**A**) Microfluidic device setup of in vitro imaging of monocyte behaviors in the vasculature network formed by the vascular endothelial cells and fibroblasts. (**B**) Three motility behaviors of monocytes in the time-lapse imaging, represented by cell 29, cell 32, and cell 40. (**C**) Speed of the three representative cells during a 25-s time window, with variations in their redox ratio showing the relation between metabolic and motility behaviors. (**D**) Trajectories of cells analyzed in the entire 150-s time window. (**E**) Statistics of the redox ratio for stationary and moving cells. Central line: median; box: interquartile range (IQR), encompassing the middle 50% of the data; outliers: individual data points falling outside 1.5 times the IQR. Scale bars, 50 μm.

To examine the capability of dSLAM for capturing faster dynamics, monocyte behaviors were tracked in vitro to mimic the immune cell recruitment process in the living vascular network (Fig. 5A and see Materials and Methods for sample preparation) (*59*), with time-lapse imaging at a pixel rate of 0.5 MHz, which permits a frame rate of 2 Hz and a field of view (FOV) of 300 μm by 210 μm for simultaneously monitoring multicellular metabolic and motility behaviors (see table S2 for details). In Fig. 5D, the metabolic activities and motility features of 50 cells were characterized over a 150-s imaging session, with captured speeds exceeding 1000 μm/min (cell 29 in Fig. 5C). We observed a few motility behavioral patterns during this window (Fig. 5B). As an example, cell 29 exhibited rapid movement within the lumen and maintained a redox ratio indicative of a high metabolic state, while cell 40 remained stationary, with a stable redox ratio of $R_{40}=0.73$. Cell 32 showed a marked decrease in both speed and redox ratio before adhering to the vasculature (see movie S3). Figure 5C illustrates the speed and metabolic changes of the three representative cells during the monocyte recruitment. To investigate the potential link between cell recruitment and metabolic activity, Fig. 5E depicts the redox ratio of stationary and moving cells tracked in Fig. 5D, suggesting a lower redox ratio in stationary cells, potentially pointing to increased metabolic activities in monocytes that have attached to the vascular networks. The application of this imaging platform to various living tissue models and pathologies could offer distinctive insights into the metabolic changes accompanying immune cell dynamics within multicellular systems.

## DISCUSSION

In summary, we have developed the dSLAM imaging platform for deep and dynamic structural and metabolic imaging by leveraging an MMF-based, high peak power, 1100-nm excitation source. We have extended the imaging depth of NAD(P)H to more than 700 μm in living engineered human microtissues, opening up possibilities for visualizing metabolic processes and structural features in thicker living tissues. This enhanced depth, combined with improved imaging speed, holds promise for new investigations into complex cellular interactions in biomedical research.

The deep imaging capabilities of dSLAM, with its four complementary label-free modalities, enable the imaging and identification of cells deep within living tissues. In the case of blood-brain barrier microfluidic devices, observed trends in optical signatures align with existing literature for different cell types. For example, endothelial cells exhibit low redox ratios due to anaerobic ATP generation via glycolysis (*27*, *51*–*54*), while astrocytes display strong THG signals in their soma (*52*, *58*). The extended imaging depth allows us to reveal heterogeneity in cell types across different depth positions. For example, higher FAD signals were observed in pericytes located in the middle of the microfluidic model, raising questions about the relationship between depth, surrounding cell compositions, and pericyte function (*55*–*57*). Further comparison of our in vitro results with in vivo studies will validate these findings and advance our understanding of the natural behavior of the human blood-brain barrier.

Cellular bioenergetics play a crucial role in many essential biological processes, including growth and proliferation, enabling cells to adapt to dynamic environments (*60*). Studying cellular bioenergetics at the cellular level has gained increasing attention due to the growing understanding of cell population heterogeneity and complex migratory behaviors (*61*). While molecular fluorescent probes [e.g., for ATP:adenosine 5′-diphosphate ratios (*62*), glucose uptake/lactate production (*63*), and NADH:NAD^+^ ratio (*64*)] have enabled substantial elucidation of metabolic pathways, dSLAM microscopy offers a label-free approach to characterize the concentration of NAD(P)H and FAD in living tissues without introducing exogenous labels. For example, glycolysis has been consistently found to be an essential metabolic pathway in macrophage migration, resulting in lower redox states of macrophages (*65*–*67*). Similar to findings in monocyte behavior, where moving cells may exhibit a higher redox ratio due to increased energy consumption and reduced oxygen availability, cancer cells have also been found to migrate with higher redox states in animal models (*62*) and cell cultures (*63*). Leveraging the adaptability of dSLAM imaging to further investigate why and how different cells use various metabolic pathways will advance our understanding of cellular bioenergetics.

The utilization of an MMF with a compact slip-on fiber shaper facilitates the flexible delivery of high peak power pulses with a near-Gaussian beam profile, offering potential for miniaturization and broader adoption compared to prior approaches. Compared to Ti:Sapphire lasers that are more commonly used for metabolic imaging, the laser system required for this setup can be a submicrojoule mode-locked Yb laser and can likely be purchased at substantially reduced cost. Compared to the original SLAM imaging implementation (*9*), which used a spatial light modulator–based pulse shaper requiring specialized broadband near-infrared optics and instrumentation expertise for optimal performance (*68*), the slip-on fiber shaper-based approach substantially increases energy conversion efficiency and substantially reduces cost. In addition, the ease of larger-core fiber alignment and fiber shaper optimization further enhances the accessibility of this label-free imaging methodology (*69*). However, hardware challenges remain to push the limits of this imaging technique. First, beam properties are not yet fully optimized compared to pulse shaper-based solutions. To leverage the power scalability of multimode fiber, further investigation into the effective modulation of multimodal nonlinear pulse propagation is needed to achieve a transform-limited pulse (*29*, *35*–*42*). Second, to enable long-term (see fig. S5), high-speed imaging, further research is required on fiber source stability (see fig. S6), scanning schemes to minimize photobleaching and photodamage (*70*, *71*) (see note S2 for estimation of saturation energy), and hardware and software codesign to reduce scattering (*72*) and enhance signal collection (*73*) (see note S3 for discussion on the speed and depth limit of dSLAM microscopy). In addition, to accurately characterize the redox ratio, potential contributions from other autofluorophores should be investigated and mitigated (*74*–*77*). For instance, lipofuscin has been reported as a potential confounder for NAD(P)H and FAD fluorescence measurements in certain biological samples (*75*). While lipofuscin is not known to be dominant in the samples imaged in this work, its potential presence should be considered for broader applicability of this technique (see table S3 for summary of known endogenous substances in biological tissues). Moreover, to enhance user-friendliness for biological studies and translational applications, efforts are needed in software development for fully automated cellular redox analysis and accurate cell segmentation that excludes nucleus (*78*, *79*), reducing the barrier to entry for nonexperts.

Advanced microscopy technologies have revolutionized our understanding of biology, each offering unique advantages for specific applications. Confocal microscopy, for instance, provides excellent resolution and optical sectioning capabilities, ideal for imaging relatively thin samples (*80*). Light-sheet microscopy enables rapid volumetric imaging of larger, live specimens, particularly after tissue clearing (*81*). Multiphoton microscopy, as exemplified by 2P and 3P imaging of labeled neurons in the mouse brain, excels in deep tissue imaging and has yielded unprecedented insights in neuroscience (*13*). Our proposed dSLAM microscopy, with its label-free, noninvasive deep tissue imaging capabilities, serves as a complementary tool for studying cellular dynamics in living tissues, requiring minimal sample preparation and no exogenous labels. With continued advancements in beam optimization, system design, and streamlined data analysis, we anticipate that dSLAM microscopy will become increasingly accessible to biomedical researchers, offering a valuable addition to the existing arsenal of microscopy techniques for investigating living tissues.

## MATERIALS AND METHODS

### MMF and beam characterizations

An SI MMF (Thorlabs, FG025LJA; 25/125 μm, 0.10 NA) of 8.5 cm in length was launched by a high-power femtosecond mode-locked ytterbium laser (Light Conversion, Carbide) at 1030 nm with a pulse energy of 350 nJ and a pulse width of 219 fs. Weak focusing was achieved using an achromatic doublet with 100-mm focal length (Thorlabs, AC254-100-B) for fiber coupling with a coupling efficiency of 76%. The output supercontinuum generation pulses were collimated by a 25.4-mm focal length off-axis parabolic mirror (Edmund Optics, 36-586) and spectrally filtered by a bandpass filter of 1100 ± 25 nm (Edmund Optics, 85-906). A continuously variable neutral density (ND) filter (Thorlabs, NDC-50C-4M-B) was placed before the imaging system to adjust the input pulse energy during imaging. For comparison, the laser reference images in Figs. 1C and 3A were collected by corresponding wavelengths using OPA (Light Conversion, Cronus-3P). The output pulse spectrum characterization was performed using an NIR spectrometer (Ocean Insight, NIRQuest+1.7). The pulse width was measured using an autocorrelator (Light Conversion, GECO). The near-field output spatial profile was measured using a complementary metal-oxide semiconductor–based camera (Mako, G-040B). The metric used to characterize beam profile in Fig. 2 was the correlation between the measured beam profile and an ideal fundamental LP_01_ intensity distribution. The mode-field diameter (MFD) of the ideal fundamental LP_01_ mode can be calculated as

$$\text{MFD}=2a\left(0.65+\frac{1.619}{V^{3/2}}+\frac{2.879}{V^{6}}\right)$$ (1)

where V=2πaNA/λ is the normalized frequency, *a* is the fiber core radius, and λ is the wavelength. The calculated MFD of the MMF used in this study was 18.2 μm.

### Fiber shaper design

The fiber shaper was built on our previous device (*29*) but with a modification to make it smaller. The fiber shaper device was driven by a single stepper motor-based actuator (Hilitand, 2-Phase 4-Wire Stepper Motor, around $25 dollars) with a microcontroller unit (Arduino, Uno Rev3, around $25), having a motion range of 34 mm and a step size of 0.025 mm. A customized fiber holder was fabricated using acrylic by a laser cutter (Universal, PLS6.75) and was assembled on the actuator slider. A microcontroller unit (Arduino, Uno Rev3) was used to control the stepper motor, with codes programmed in Python and sent from a personal computer (PC). To adapt to the short fiber length for minimizing pulse dispersion, a single high-precision actuator was used, with a minimal bending radius of 5 mm.

### SLAM imaging system

The SLAM microscopy was implemented as an inverted scanning microscope. A galvanometer mirror pair (ScannerMAX, Saturn-5 Galvo and Saturn-9 Galvo) was used to raster scan the 1100-nm beam out of the MMF. A water immersion objective (Olympus, XLPLN25XWMP2) focused the beam on the imaging plane. A microscope stage (ASI, MS2000) was used to adjust the center of the FOV for mosaic scanning and the focal plane for deep imaging series. The excitation and emission paths were separated by a dichroic mirror (Thorlabs, DMLP650L). The emission signals were further separated into four detection channels using dichroic mirrors (Chroma, T412lpxt, T505lpxr, T570lpxr) and bandpass filters (Chroma, ZET365/20x; Edmund Optics, 84-095; Edmund Optics, 65-159; Semrock, FF01-609/57-25), corresponding to THG, NAD(P)H, SHG, and FAD. Photons were collected by four individual photomultiplier tubes (Hamamatsu, H16201), and signals were translated to images through a custom-written LabVIEW acquisition software. The individual images were colored and merged using the Fiji software (*50*). The pseudo-colors for the four modalities were consistently chosen as magenta hot for THG, cyan hot for NAD(P)H, green for SHG, and yellow hot for FAD throughout this study. A commercial OPA (Light Conversion, Cronus-3P) emitting pulses at 750 nm (approximately 40 fs) and 1300 nm (46 fs) was also directed to the same microscope system for acquiring reference images.

### Volumetric imaging

3D volume images were generated by stacking 2D images acquired at various depths and visualized using the “3D viewer” in the Fiji software (*50*). Depth-resolved 2D images were acquired by programmatically moving the sample stage (ASI, MS2000) vertically from superficial layers (Z=0 μm) to deeper layers with fixed step size. Pulse energy at the excitation focus was maintained constant in each imaging plane by adjusting an ND filter (Thorlabs, NDC-50C-4M-B) placed at the fiber output end to compensate for optical attenuation in deeper layers. The pulse energy $E_{Z}$ at the excitation focus of depth *Z* was estimated from the pulse energy at the sample surface $E_{0}$ by $E_{Z}=E_{0}\text{exp}\left(-Z\ell_{e}\right)$, with the EAL $\ell_{e}$ of the samples precalibrated (*15*).

### Image analysis

Raw 16-bit images were presented without denoising or deconvolution algorithm applied in the data visualization in this study. Same contrasts were applied to compare signal levels in Fig. 2A (initial versus optimized) and Fig. 3A (2PAF versus 3PAF). Imaging SNR was calculated as the average value of the brightest 0.5% pixels (*15*). Imaging SBR was calculated as the ratio of the average value of the brightest 0.5% pixels and the average value of the pixels in the background mask. Imaging resolution was characterized by imaging 0.1-μm green and 0.2-μm blue subdiffraction-limited fluorescent beads (BangsLabs, FCDG002, FCGB003), which were fitted with a 2D Gaussian distribution in both lateral and axial planes. The full width at half maximum of the fitted distribution was used to represent the resolution.

### Measurements of redox ratio

The optical redox ratio is defined as the ratio of the concentrations of FAD and NAD(P)H

$$R=\frac{\text{FAD}}{\text{FAD}+\text{NAD}\left(P\right)H}$$ (2)

The relationship between pixel values in the image and the actual concentrations of FAD and NAD(P)H was calibrated using 1 mM standard FAD (MilliporeSigma, F6625) and NAD(P)H (MilliporeSigma, N8129) solutions. The calibration accounted for multiphoton generation efficiency, system collection efficiency, and PMT gain. For the redox map in Fig. 4D, the pixel-wise redox ratio was calculated using a manually created cell mask that excluded the background and nuclei. For the cellular redox analysis in Figs. 4 (G and I) and 5 (C and E), the redox ratio was calculated using the average concentration within individual cells. These cells were automatically segmented on the basis of pixel intensity values using the “mean” method in Fiji software (code S1) (*50*). In Fig. 4, different cell types were further manually classified (fig. S4). Endothelial cells were identified by morphology, while pericytes and astrocytes were classified using a normalized THG intensity threshold of 0.5. In Fig. 5, cell tracing analysis was performed by locating the center of moving cells in each frame and plotting the time-lapse trace. A rolling average was applied to minimize errors in manual labeling.

### Sample preparation

All animal procedures were conducted in accordance with a protocol approved by the Institutional Animal Care and Use Committee at the Massachusetts Institute of Technology.

In Figs. 1 and 2, whisker pad tissues were obtained from approximately 12-week-old adult C57BL/6 mice. Each mouse received an intraperitoneal injection of heparin (1000 U/kg body weight) for anticoagulation. Following euthanasia with an isoflurane overdose, mice were transcardially perfused with ice-cold 10% sucrose in deionized water to thoroughly rinse blood vessels. The whisker pad was then carefully dissected from underlying structures of masseter muscle and nasal bone using blunt dissection. Approximately 100 μm of the top skin layer was shaved off, and the tissues were fixed in 4% paraformaldehyde solution at 4°C for repeated use.

In Fig. 4, the microvascular network of the blood-brain barrier was engineered using a triculture of primary human astrocytes (ScienCell, 1800), primary human brain pericytes (ScienCell, 1200), and induced pluripotent stem cell–derived vascular endothelial cells (Alstem, iPS11). Cells were expanded using the following media: astrocyte medium (ScienCell, 1801), pericyte medium (ScienCell, 1201), and VascuLife VEGF endothelial medium (Lifeline Cell Technology, LL-0003) supplemented with 10% fetal bovine serum (Thermo Fisher Scientific, 26140-079) and SB 431542 (Selleckchem, S1067). Cells were passaged using TrypLE Express (Thermo Fisher Scientific, 12604021). The 3D microfluidic coculture was conducted as described in (*82*). For Fig. 4C, IF staining for anti-platelet–derived growth factor receptor beta (Abcam, ab69506; RRID: AB1269704) and anti-glial fibrillary acidic protein (Abcam, ab10062; RRID: AB296804) was conducted as described in (*49*).

In Fig. 5, human umbilical vein endothelial cells (HUVECs; Angioproteomie, cAP-0001) and normal human lung fibroblasts (NHLFs; Lonza, CC-2512) were cultured in VascuLife VEGF endothelial medium and FibroLife S2 fibroblast medium (Lifeline Cell Technology, LL-0011), respectively. After the cells were detached with TrypLE Express, they were suspended in a thrombin solution, mixed with fibrinogen, and seeded into a microfluidic device, as previously described (*59*). The cell-laden fibrin mix (10 μl) was seeded per device into a central channel that measured approximately 9 mm by 3 mm by 500 μm, with final cell concentrations of 7 and 1.5 M/ml for the HUVECs and NHLFs, respectively. Devices were kept in the incubator after gelation, with daily media changes of VascuLife medium for 6 days until the HUVECs self-assembled into perfusable microvascular networks. Primary human monocytes were isolated from buffy coats acquired from Massachusetts General Hospital. Monocytes were resuspended at 2 M/ml before addition to microvascular networks.

## Supplementary Materials

### The PDF file includes:

Supplementary Notes S1 to S3 Figs. S1 to S6 Tables S1 to S3 Legend for code S1 Legends for movies S1 to S3 References

### Other Supplementary Material for this manuscript includes the following:

Code S1
