# Supplementary material for: Deep and dynamic metabolic and structural imaging in living tissues
Source: Sci Adv. 2024 Dec 11;10(50):eadp2438. doi: 10.1126/sciadv.adp2438 (PMC11633739; doi:10.1126/sciadv.adp2438)
Supplement: Supplementary file 1 — Supplementary Notes S1 to S3 Figs. S1 to S6 Tables S1 to S3 Legend for code S1 Legends for movies S1 to S3 References [file sciadv.adp2438_sm.pdf]

Science Advances
AAAS

Supplementary Materials for

Deep and dynamic metabolic and structural imaging in living tissues

Kunzan Liu *et al.*

Corresponding author: Tong Qiu, qiutong@mit.edu; Sixian You, sixian@mit.edu

Sci. Adv. **10**, eadp2438 (2024)
DOI: 10.1126/sciadv.adp2438

The PDF file includes:

Supplementary Notes S1 to S3
Figs. S1 to S6
Tables S1 to S3
Legend for code S1
Legends for movies S1 to S3
References

Other Supplementary Material for this manuscript includes the following:

Code S1
Movies S1 to S3

Supplementary Note 1 Estimation of 3P action cross section of NAD(P)H at 1100 nm.

To estimate the 3P action cross section (absorption cross section $\sigma^{(3)}$ multiplied by the fluorescence quantum yield η) of NAD(P)H, we used 3PAF and 2PAF NAD(P)H imaging signals as the indicators of the time-averaged fluorescence photon fluxes. Specifically, time-averaged fluorescence photon fluxes, $S_{2\mathrm{P}}$ and $S_{3\mathrm{P}}$, can be calculated as (*45, 83*)

$$S_{2\mathrm{P}} = \frac{1}{2}\frac{g_p^{(2)}}{f\tau^{(2)}}\phi\eta\sigma^{(2)}Cn_0\frac{a^{(2)}}{8\pi\lambda^{(2)}}\left(\frac{E^{(2)}}{hc/\lambda^{(2)}}\right)^2, \tag{S1}$$

$$S_{3\mathrm{P}} = \frac{1}{3}\frac{g_p^{(3)}}{(f\tau^{(3)})^2}\phi\eta\sigma^{(3)}Cn_0\frac{a^{(3)}\mathrm{NA}^2}{8\lambda^{(3)3}}\left(\frac{E^{(3)}}{hc/\lambda^{(3)}}\right)^3, \tag{S2}$$

where $g_p^{(n)}$ is the n-th order temporal coherence of the excitation source (assume Gaussian pulse with $g_p^{(2)} = 0.66$ and $g_p^{(3)} = 0.51$), f is the laser repetition rate, $\tau^{(n)}$ is the pulse duration at the excitation focus, ϕ is the collection efficiency of the system, η is the fluorescence quantum yield, $\sigma^{(n)}$ is the n-photon absorption cross section, C is the sample concentration, n_0 is the refractive index of the sample medium, $a^{(n)}$ is a constant with $a^{(2)} = 64$ and $a^{(3)} = 28.1$, $\lambda^{(n)}$ is the n-photon excitation wavelength, $E^{(n)}$ is the excitation pulse energy, $h = 6.626\times10^{-34}$ J·s is the Planck's constant, and $c = 3\times10^8$ m/s is the speed of light in vacuum.

We performed 2PAF and 3PAF imaging using $\lambda^{(2)}$ =750 nm and $\lambda^{(3)}$ =1100 nm, respectively, at the same site of the microvascular network in Fig. 3. We recorded the excitation pulse energy $E^{(2)} = 0.8$ nJ and $E^{(3)} = 3.0$ nJ that achieved the same SNR of the image ($S_{2\mathrm{P}} = S_{3\mathrm{P}}$), which can be used to estimate the 3P action cross section $\eta\sigma^{(3)}$ of NAD(P)H, given (*84*)

$$\eta\sigma^{(2)} \approx 2.0\times10^{-52}\,\mathrm{cm}^4\cdot\mathrm{s}\cdot\mathrm{photon}^{-1}. \tag{S3}$$

We obtained 3P action cross section

$$\eta\sigma^{(3)} \approx 2.3\times10^{-84}\,\mathrm{cm}^6\cdot\mathrm{s}^2\cdot\mathrm{photon}^{-2}, \tag{S4}$$

with the estimations of $\tau^{(2)} = \tau^{(3)} = 150$ fs at the excitation focus. The order of magnitude is consistent with expectations based on the single-intermediate-state (SIS) approximation (*44*). However, future efforts should focus on obtaining a more accurate estimation of the 3P action cross section over a wider spectral range, which will require a more sophisticated experimental setup (*83, 85*).

Supplementary Note 2 Estimation of saturation pulse energy for 3PAF NAD(P)H imaging.

The saturation pulse energy is defined as the energy $E_{\rm sat}^{(n)}$ that corresponds to a n-photon excitation probability of 0.63, which satisfies (*15, 86*)

$$E_{\rm sat}^{(2)} = \frac{{\lambda^{(2)}}^2}{\pi {\rm NA}^2} \sqrt{\frac{\tau^{(2)}}{g_p^{(2)}\sigma^{(2)}}} \cdot \frac{hc}{\lambda^{(2)}}, \tag{S5}$$

$$E_{\rm sat}^{(3)} = \frac{{\lambda^{(3)}}^2}{\pi {\rm NA}^2} \sqrt[3]{\frac{{\tau^{(3)}}^2}{g_p^{(3)}\sigma^{(3)}}} \cdot \frac{hc}{\lambda^{(3)}}, \tag{S6}$$

with the same definition of parameters in Supplementary Note 1.

With system NA= 1.05 and the parameters in Supplementary Note 1, we estimate the saturation energy for 2PAF and 3PAF imaging using $\lambda^{(2)}$ =750 nm and $\lambda^{(3)}$ =1100 nm, respectively, as

$$E_{\rm sat}^{(2)} \approx 14.5\,{\rm nJ}, \quad E_{\rm sat}^{(3)} \approx 16.9\,{\rm nJ}. \tag{S7}$$

In imaging, to minimize photobleaching and photodamage, the pulse energy $E_{\rm focus}^{(n)}$ at the excitation focus should be substantially below this saturation energy $E_{\rm sat}^{(n)}$ (e.g., $E_{\rm focus}^{(n)} < 0.3E_{\rm sat}^{(n)}$ (*86*). The pulse energy at the excitation focus used in this work was

$$E_{\rm focus}^{(2)} = 0.8\,{\rm nJ}, \quad E_{\rm focus}^{(3)} = 3.0\,{\rm nJ} \tag{S8}$$

for 2PAF and 3PAF NAD(P)H imaging, respectively, which satisfied the requirement. Supplementary Fig. S5 also demonstrated that no photodamage was observed during repeated imaging within a specific time period. However, factors such as sample type, fluorophore, excitation wavelength, and imaging conditions (*in vivo*/*in vitro*) can all influence the extent of photodamage and photobleaching, which requires further investigations (*86*).

Supplementary Note 3 Discussion on the speed and depth limit of dSLAM microscopy.

Speed Limit: In this work, the highest frame rate achieved was 2 Hz for a field-of-view of 300 µm×210 µm (252,000 pixels), with a laser repetition rate of 0.5 MHz, which was found to be optimal for minimizing bleaching and damage. We observed that this repetition rate was optimal for minimizing bleaching and damage, aligning with existing work demonstrating that photobleaching and damage, potentially exacerbated by triplet state population, can be mitigated by using low repetition rate excitation (<0.5∼1 MHz) (*87*). Further joint optimization of scanning routes, repetition rate, and heat dissipation may lead to improvements in this regard.

Depth limit: The depth we achieved in this work was 720 µm for the living blood-brain barrier model, limited by the depth of the sample. In theory, the imaging depth for achieving a certain SBR would be determined by the sufficient number of photons arriving at the excitation focus after experiencing absorption and scattering (*86*). The number of ballistic photons reaching depth z is proportional to the peak power P_z at the excitation focus, expressed as

$$P_z = P_0 \exp\left(-\frac{z}{\ell_e}\right) = P_0 \exp\left(-\frac{z}{\ell_a} - \frac{z}{\ell_s}\right), \tag{S9}$$

where P_0 is the peak power at the sample surface, ℓ_e is the effective attenuation length (Fig. 3E) that is contributed from the water absorption length ℓ_a and the scattering mean-free path ℓ_s (*13*). One potential factor restricting this surface power P_0 is tissue heating, as reported in (*15*), where the surface average power could not exceed 80 mW at 1300 nm for lymph node imaging. In this work, we found that 15 mW at 1100 nm is permissible for long-term, repeated deep imaging of living engineered human multicellular microtissues. Therefore, the imaging depth is primarily limited by the permissible power P_0 at the sample surface. It is important to note that this is a theoretical model assuming uniform and consistent scattering coefficients. For dSLAM imaging in more general conditions, the actual depth limit may also depend on sample type (organ, *in vivo*/*ex vivo*) and acquisition parameters (FOV, pixel dwell time), necessitating further investigation.

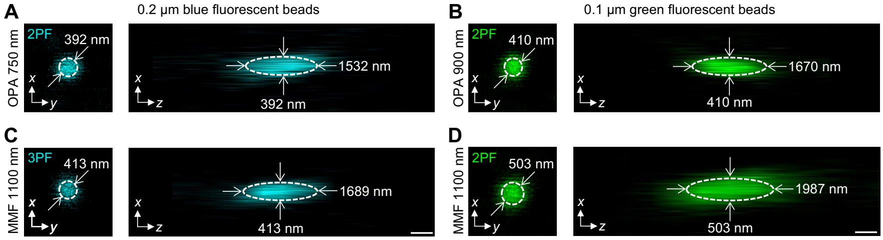

Fig. S1 Measurements of imaging resolution using fluorescent beads. Resolution was estimated using the FWHM of the 0.1 µm green and 0.2 µm blue sub-diffraction-limited fluorescent beads (BangsLabs, FCDG002, FCGB003). (**A**) OPA 750 nm for 2PF. (**B**), OPA 900 nm for 2PF. (**C**) MMF 1100 nm for 3PF. (**D**) MMF 1100 nm for 2PF. Scale bars: 0.5 µm.

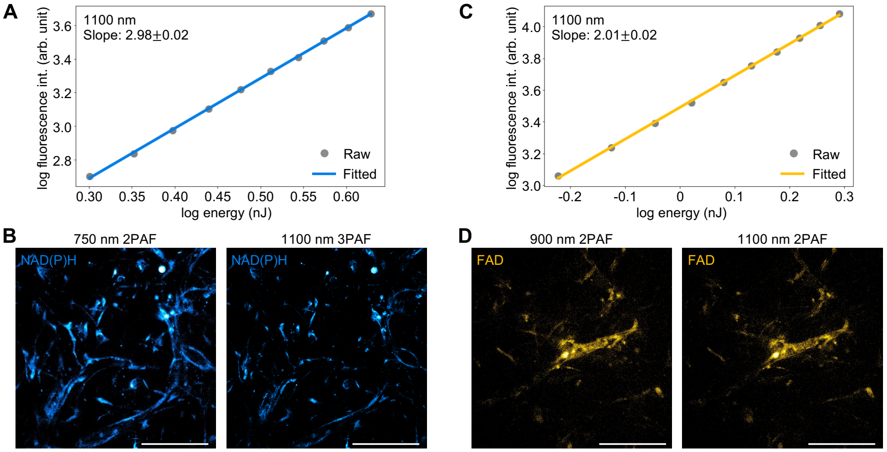

Fig. S2 Validation of metabolic imaging using 1100 nm excitation. (**A**) Signal dependence on energy of NAD(P)H measured with NAD(P)H solution. (**B**) Co-registered NAD(P)H images acquired with 750 nm (OPA, 2PAF) and 1100 nm (MMF, 3PAF). (**C**) Signal dependence on energy of FAD measured with FAD solution. (**D**) Co-registered FAD images acquired with 900 nm (OPA, 2PAF) and 1100 nm (MMF, 2PAF). The energy was measured at the sample surface, and the fluorescence intensity was calculated using the SNR of the image of the solution. Scale bars: 100 µm.

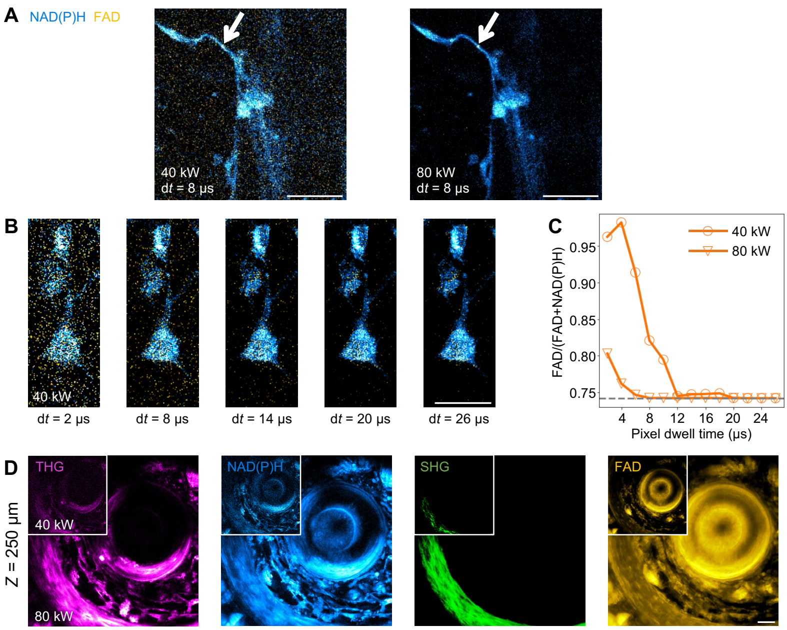

Fig. S3 High-peak-power sources for deep and dynamic imaging. (**A**) Comparison of images (blood-brain barrier microfluidic model) acquired with peak power of 40 kW and 80 kW with the same pixel dwell time. (**B**) Comparison of images (blood-brain barrier microfluidic model) acquired with peak power of 40 kW with increased pixel dwell time. (**C**) Calculated redox ratio with different peak power and pixel dwell time. (**D**) Comparison of images (mouse whisker pad) acquired with peak power of 40 kW and 80 kW at the depth of Z=250 μm. Scale bars: 30 μm.

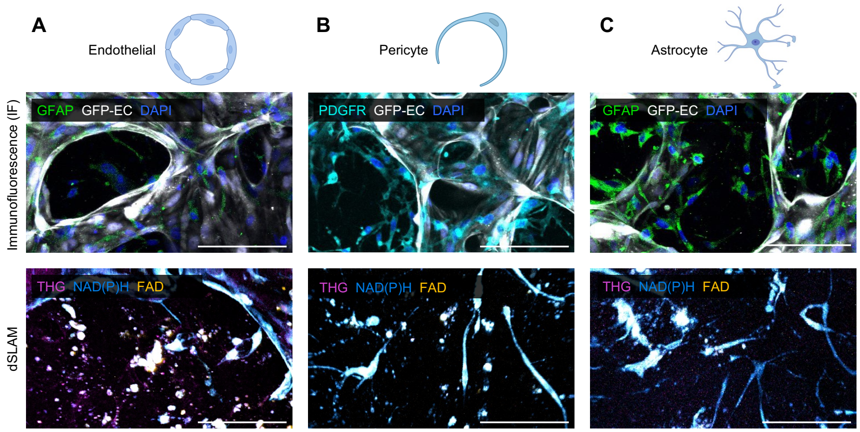

Fig. S4 Validation of cell types in human blood-brain barrier microfluidic model. Immunofluorescence (IF) images and dSLAM images of endothelial cells (**A**), pericytes (**B**), and astrocytes (**C**). dSLAM images were acquired using three individual microfluidic models, each containing only one cell type. Scale bars: 100 μm.

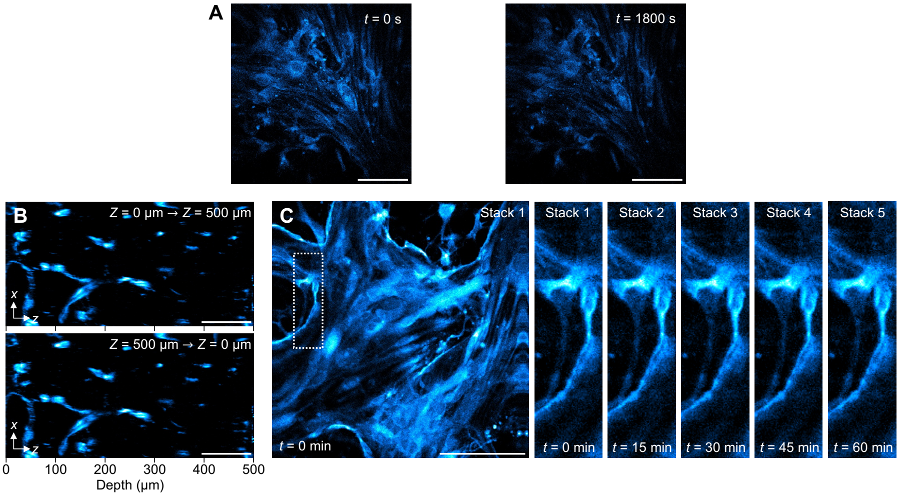

Fig. S5 Investigations on repeated dSLAM imaging. (**A**) Comparison of repeated dynamic imaging for 1800 s. (**B**) Comparison of volumetric imaging from the shallowest to the deepest layers (from Z=0 µm to Z=500 µm) and from the deepest layers to the shallowest layers (from Z=500 µm to Z=0 µm). (**C**) Comparison of repeated volumetric imaging for the same stack of the entire depth of 500 µm. Scale bars: 100 µm.

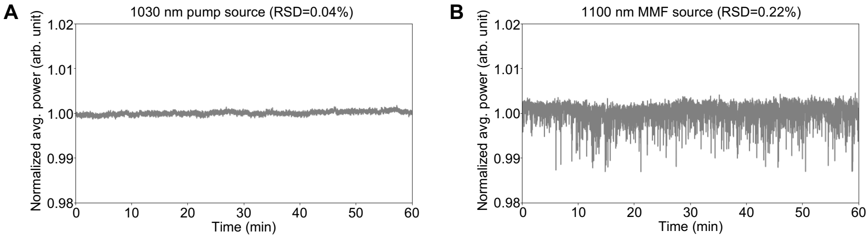

Fig. S6 Source stability. Normalized average power of the 1030 nm pump source (**A**) and the 1100 nm MMF source (**B**) recorded through a silicon-based photodiode sensor (Thorlabs PM16-130) per 0.2 s for a period of 60 min. A pulse energy of 350 nJ was used to pump the MMF, consistent with the imaging conditions. RSD: relative standard deviation.

Supplementary Table S1 Summary of instrumentations and parameters of NAD(P)H imaging in existing works.

Ref.	Excitation wavelength [nm]	Laser	Laser power	Rep. rate [MHz]	Deepest depth reported [µm]	Pixel dwell time [µs]	Resolution [µm]	Microscope system	Objective lens
(*8*)	750	Insight DS+ (Spectra Physics)	3.0 ∼3.2 mW at the sample	-	-	4.6	-	Ultima (Bruker)	×100, NA 1.45 objective (Nikon Plan Apo Lambda)
(*4*)	800	Ti:sapphire laser (Spectra Physics, Tsunami)	15 mW at the sample	-	70	11.5	-	Inverted microscope (Nikon, Eclipse TE300)	×40, NA 1.3, oil-immersion objective (Nikon PlanApo)
(*88*)	755	Insight femtosecond laser	10 ∼60 mW at the sample surface	-	120	0.4	-	Leica SP8 inverted microscope	×40, NA 1.1, water-immersion objective
(*6*)	835	Chameleon ultra-tunable ultra-fast laser (Coherent)	40 mW after the objective	76	100	1.4 ∼ 3.7		Laser-scanning microscope (Zeiss LSM510 -Axioskop-2)	×40, NA 0.80 objective
(*7*)	750	Ultrafast femtosecond laser (InSight DS+, Spectra Physics)	-	-	75	-	-	Custom-built multi-photon microscope (Bruker)	×40, NA 1.13, water-immersion objective (Nikon)
(*11*)	730 ∼ 900	Ti:sapphire laser (Millenia-Tsunami combination, Spectra Physics)	3.1 ∼10.5 mW at the sample	80	-	0.6	-	Zeiss Axiovert-135 inverted microscope (Carl Zeiss)	×40, NA 1.3, oil-immersion objective (Zeiss F Fluar)
(*9*)	1110	Customized PCF source with pulse shaper (pumped by femtoTrain, Spectra Physics)	14 mW at the sample	10	115	5 ∼ 12	0.7	Custom-built inverted scanning microscope	×40, NA 1.15 objective (UAPON 40XW340, Olympus)

Supplementary Table S2 Summary of detail information in image acquisition.

Figure	Sample	FOV /volume [µm]	Pixel/voxel size [µm]	Pixel dwell time [µs]	Imaging depth [µm]	Average power at sample surface [mW]	Peak power at sample surface [kW]	Rep. rate [MHz]
1C (900 nm)	Fixed mouse whisker pads (ex vivo)	(373, 373)	(0.4, 0.4)	4	150	1.6	16	1
1C (1300 nm)	Fixed mouse whisker pads (ex vivo)	(373, 373)	(0.4, 0.4)	4	150	6.0	60	1
1C (1100 nm)	Fixed mouse whisker pads (ex vivo)	(373, 373)	(0.4, 0.4)	4	150	6.0	60	1
2A (initial)	Fixed mouse whisker pads (ex vivo)	(373, 373)	(0.4, 0.4)	4	150	4.8	48	1
2A (optimized)	Fixed mouse whisker pads (ex vivo)	(373, 373)	(0.4, 0.4)	4	150	6.0	60	1
3 (1100 nm)	Fixed microvascular network (in vitro) (*46, 47*)	(400, 400, 720)	(0.67, 0.67, 3)	6	0∼720	1.5∼15	10∼100	1
3 (750 nm)	Fixed microvascular network (in vitro) (*46, 47*)	(400, 400, 400)	(0.67, 0.67, 3)	6	0∼400	0.3∼3	2∼20	1
4	Living and intact human blood-brain barrier microfludic model (in vitro) (*49*)	(350, 350, 500)	(0.5, 0.5, 2)	6	0∼500	1.5∼15	10∼100	1
5	Living vascular network with monocytes (in vitro) (*59*)	(300, 210)	(0.5, 0.5)	2	20	3.0	60	0.5

Supplementary Table S3 Summary of label-free multiphoton imaging of endogenous substances in biology.

Endogenous substances	1-photon excitation peak [nm]	Emission (at half maximum) [nm]	Typical excitation wavelength	Tissue type
NAD(P)H	262<br>352	417∼491	2PAF 740∼750 nm (*84*)<br>2PAF 700 nm (*20*)<br>2PAF 730∼800 nm (*23*)<br>2PAF 700∼750 nm (*11*)<br>2PAF 800 nm (*4*)	Cells
FAD	260<br>387<br>456	521∼586	2PAF 700∼975 nm (*11*)<br>2PAF 860 nm (*20*)<br>2PAF 890 nm (*4*)	Cells
Elastin	350	368∼483	2PAF 740∼750 nm (*84*)<br>2PAF 860 nm (*20*)	Human skin (*84*),<br>mouse arteriole (*84*),<br>mouse lung (*20*)
Lipofuscin	344	521∼586	2PAF 740 nm (*84*)	Human neurofibrillary tangle (*84*)
Porphyrins	400	620∼642	-	-
Pyridoxine	311	372∼432	-	-
Tryptophan	215<br>273	309∼373	3PAF 750 nm (*84*)	Mouse choroid plexus,<br>mouse small intestine (*84*)

Supplementary Code 1 Cell segmentation code for Fiji software.

Supplementary Video 1 Imaging data for deep metabolic and structural imaging in the intact living human blood-brain barrier microfluidic model.

Supplementary Video 2 Rendered 3D imaging for deep metabolic and structural imaging in the intact living human blood-brain barrier microfluidic model.

Supplementary Video 3 Imaging data for dynamic metabolic and structural imaging of monocyte behaviors *in vitro*.

REFERENCES AND NOTES

1. I. Georgakoudi, B. C. Jacobson, M. G. Muller, E. E. Sheets, K. Badizadegan, D. L. Carr-Locke, C. P. Crum, C. W. Boone, R. R. Dasari, J. Van Dam, M. S. Feld, NAD(P)H and collagen as in vivo quantitative fluorescent biomarkers of epithelial precancerous changes. *Cancer Res.* **62**, 682–687 (2002).

2. O. I. Kolenc, K. P. Quinn, Evaluating cell metabolism through autofluorescence imaging of NAD(P)H and FAD. *Antioxid. Redox Signal.* **30**, 875–889 (2019).

3. Z. Liu, D. Pouli, C. A. Alonzo, A. Varone, S. Karaliota, K. P. Quinn, K. Münger, K. P. Karalis, I. Georgakoudi, Mapping metabolic changes by noninvasive, multiparametric, high-resolution imaging using endogenous contrast. *Sci. Adv.* **4**, eaap9302 (2018).

4. M. C. Skala, K. M. Riching, A. Gendron-Fitzpatrick, J. Eickhoff, K. W. Eliceiri, J. G. White, N. Ramanujam, In vivo multiphoton microscopy of NADH and FAD redox states, fluorescence lifetimes, and cellular morphology in precancerous epithelia. *Proc. Natl. Acad. Sci. U.S.A.* **104**, 19494–19499 (2007).

5. A. J. Walsh, R. S. Cook, H. C. Manning, D. J. Hicks, A. Lafontant, C. L. Arteaga, M. C. Skala, Optical metabolic imaging identifies glycolytic levels, subtypes, and early-treatment response in breast cancer. *Cancer Res.* **73**, 6164–6174 (2013).

6. G. R. J. Gordon, H. B. Choi, R. L. Rungta, G. C. R. Ellis-Davies, B. A. MacVicar, Brain metabolism dictates the polarity of astrocyte control over arterioles. *Nature* **456**, 745–749 (2008).

7. T. M. Heaster, A. R. Heaton, P. M. Sondel, M. C. Skala, Intravital metabolic autofluorescence imaging captures macrophage heterogeneity across normal and cancerous tissue. *Front. Bioeng. Biotechnol.* **9**, 644648 (2021).

8. A. J. Walsh, K. P. Mueller, K. Tweed, I. Jones, C. M. Walsh, N. J. Piscopo, N. M. Niemi, D. J. Pagliarini, K. Saha, M. C. Skala, Classification of T-cell activation via autofluorescence lifetime imaging. *Nat. Biomed. Eng.* **5**, 77–88 (2021).

9. S. You, H. Tu, E. J. Chaney, Y. Sun, Y. Zhao, A. J. Bower, Y.-Z. Liu, M. Marjanovic, S. Sinha, Y. Pu, S. A. Boppart, Intravital imaging by simultaneous label-free autofluorescence-multiharmonic microscopy. *Nat. Commun.* **9**, 2125 (2018).

10. F. Helmchen, W. Denk, Deep tissue two-photon microscopy. *Nat. Methods* **2**, 932–940 (2005).

11. S. Huang, A. A. Heikal, W. W. Webb, Two-photon fluorescence spectroscopy and microscopy of NAD(P)H and flavoprotein. *Biophys. J.* **82**, 2811–2825 (2002).

12. C. Xu, R. M. Williams, W. Zipfel, W. W. Webb, Multiphoton excitation cross-sections of molecular fluorophores. *Bioimaging* **4**, 198–207 (1996).

13. N. G. Horton, K. Wang, D. Kobat, C. G. Clark, F. W. Wise, C. B. Schaffer, C. Xu, In vivo three-photon microscopy of subcortical structures within an intact mouse brain. *Nat. Photonics* **7**, 205–209 (2013).

14. T. Wang, C. Xu, Three-photon neuronal imaging in deep mouse brain. *Optica* **7**, 947–960 (2020).

15. K. Choe, Y. Hontani, T. Wang, E. Hebert, D. G. Ouzounov, K. Lai, A. Singh, W. Béguelin, A. M. Melnick, C. Xu, Intravital three-photon microscopy allows visualization over the entire depth of mouse lymph nodes. *Nat. Immunol.* **23**, 330–340 (2022).

16. T. Wang, D. G. Ouzounov, C. Wu, N. G. Horton, B. Zhang, C.-H. Wu, Y. Zhang, M. J. Schnitzer, C. Xu, Three-photon imaging of mouse brain structure and function through the intact skull. *Nat. Methods* **15**, 789–792 (2018).

17. G. Wang, L. Li, X. Liao, S. Wang, J. Mitchell, R. C. Rabel, S. Luo, J. Shi, J. E. Sorrells, R. R. Iyer, E. Aksamitiene, C. A. Renteria, E. J. Chaney, D. J. Milner, M. B. Wheeler, M. U. Gillette, A. Schwing, J. Chen, H. Tu, Supercontinuum intrinsic fluorescence imaging heralds 'free view' of living systems. bioRxiv 577383 [Preprint] (2024). https://doi.org/10.1101/2024.01.26.577383.

18. G. Wang, L. Li, J. E. Sorrells, J. Chen, H. Tu, Gentle label-free nonlinear optical imaging relaxes linear-absorption-mediated triplet. bioRxiv 561579 [Preprint] (2023). https://doi.org/10.1101/2023.10.09.561579.

19. B. Weigelin, G.-J. Bakker, P. Friedl, Third harmonic generation microscopy of cells and tissue organization. *J. Cell Sci.* **129**, 245–255 (2016).

20. D. Débarre, W. Supatto, A.-M. Pena, A. Fabre, T. Tordjmann, L. Combettes, M.-C. Schanne-Klein, E. Beaurepaire, Imaging lipid bodies in cells and tissues using third-harmonic generation microscopy. *Nat. Methods* **3**, 47–53 (2006).

21. M. Yildirim, C. Delepine, D. Feldman, V. A. Pham, S. Chou, J. Ip, A. Nott, L.-H. Tsai, G.-L. Ming, P. T. C. So, M. Sur, Label-free three-photon imaging of intact human cerebral organoids for tracking early events in brain development and deficits in Rett syndrome. *eLife* **11**, e78079 (2022).

22. P. J. Campagnola, L. M. Loew, Second-harmonic imaging microscopy for visualizing biomolecular arrays in cells, tissues and organisms. *Nat. Biotechnol.* **21**, 1356–1360 (2003).

23. A. Zoumi, A. Yeh, B. J. Tromberg, Imaging cells and extracellular matrix in vivo by using second-harmonic generation and two-photon excited fluorescence. *Proc. Natl. Acad. Sci. U.S.A.* **99**, 11014–11019 (2002).

24. S. You, Y. Sun, E. J. Chaney, Y. Zhao, J. Chen, S. A. Boppart, H. Tu, Slide-free virtual histochemistry (Part II): Detection of field cancerization. *Biomed. Opt. Express* **9**, 5253–5268 (2018).

25. S. You, R. Barkalifa, E. J. Chaney, H. Tu, J. Park, J. E. Sorrells, Y. Sun, Y.-Z. Liu, L. Yang, D. Z. Chen, M. Marjanovic, S. Sinha, S. A. Boppart, Label-free visualization and characterization of extracellular vesicles in breast cancer. *Proc. Natl. Acad. Sci. U.S.A.* **116**, 24012–24018 (2019).

26. S. You, Y. Sun, L. Yang, J. Park, H. Tu, M. Marjanovic, S. Sinha, S. A. Boppart, Real-time intraoperative diagnosis by deep neural network driven multiphoton virtual histology. *NPJ Precis. Oncol.* **3**, 33 (2019).

27. S. You, E. J. Chaney, H. Tu, Y. Sun, S. Sinha, S. A. Boppart, Label-free deep profiling of the tumor microenvironment. *Cancer Res.* **81**, 2534–2544 (2021).

28. J. Park, J. E. Sorrells, E. J. Chaney, A. M. Abdelrahman, J. A. Yonkus, J. L. Leiting, H. Nelson, J. J. Harrington, E. Aksamitiene, M. Marjanovic, P. D. Groves, C. Bushell, M. J. Truty, S. A. Boppart, In vivo label-free optical signatures of chemotherapy response in human pancreatic ductal adenocarcinoma patient-derived xenografts. *Commun. Biol.* **6**, 980 (2023).

29. T. Qiu, H. Cao, K. Liu, L.-Y. Yu, M. Levy, E. Lendaro, F. Wang, S. You, Spectral-temporal-spatial customization via modulating multimodal nonlinear pulse propagation. *Nat. Commun.* **15**, 2031 (2024).

30. C. Xu, F. Wise, Recent advances in fibre lasers for nonlinear microscopy. *Nat. Photonics* **7**, 875–882 (2013).

31. K. Alhallak, L. G. Rebello, T. J. Muldoon, K. P. Quinn, N. Rajaram, Optical redox ratio identifies metastatic potential-dependent changes in breast cancer cell metabolism. *Biomed. Opt. Express* **7**, 4364–4374 (2016).

32. V. Miskolci, K. E. Tweed, M. R. Lasarev, E. C. Britt, A. J. Walsh, L. J. Zimmerman, C. E. McDougal, M. R. Cronan, J. Fan, J.-D. Sauer, M. C. Skala, A. Huttenlocher, In vivo fluorescence lifetime imaging of macrophage intracellular metabolism during wound responses in zebrafish. *eLife* **11**, e66080 (2022).

33. J. T. Sharick, C. M. Walsh, C. M. Sprackling, C. A. Pasch, D. L. Pham, K. Esbona, A. Choudhary, R. Garcia-Valera, M. E. Burkard, S. M. McGregor, K. A. Matkowskyj, A. A. Parikh, I. M. Meszoely, M. C. Kelley, S. Tsai, D. A. Deming, M. C. Skala, Metabolic heterogeneity in patient tumor-derived organoids by primary site and drug treatment. *Front. Oncol.* **10**, 553 (2020).

34. V. Liaudanskaya, N. J. Fiore, Y. Zhang, Y. Milton, M. F. Kelly, M. Coe, A. Barreiro, V. K. Rose, M. R. Shapiro, A. S. Mullis, A. Shevzov-Zebrun, M. Blurton-Jones, M. J. Whalen, A. J. Symes, I. Georgakoudi, T. J. F. Nieland, D. L. Kaplan, Mitochondria dysregulation contributes to secondary neurodegeneration progression post-contusion injury in human 3D in vitro triculture brain tissue model. *Cell Death Dis.* **14**, 496 (2023).

35. L. G. Wright, W. H. Renninger, D. N. Christodoulides, F. W. Wise, Nonlinear multimode photonics: Nonlinear optics with many degrees of freedom. *Optica* **9**, 824–841 (2022).

36. K. Krupa, A. Tonello, A. Barthélémy, T. Mansuryan, V. Couderc, G. Millot, P. Grelu, D. Modotto, S. A. Babin, S. Wabnitz, Multimode nonlinear fiber optics, a spatiotemporal avenue. *APL Photonics* **4**, 110901 (2019).

37. L. G. Wright, F. O. Wu, D. N. Christodoulides, F. W. Wise, Physics of highly multimode nonlinear optical systems. *Nat. Phys.* **18**, 1018–1030 (2022).

38. L. G. Wright, D. N. Christodoulides, F. W. Wise, Spatiotemporal mode-locking in multimode fiber lasers. *Science* **358**, 94–97 (2017).

39. X. Wei, J. C. Jing, Y. Shen, L. V. Wang, Harnessing a multi-dimensional fibre laser using genetic wavefront shaping. *Light Sci. Appl.* **9**, 149 (2020).

40. Y. Ding, X. Xiao, K. Liu, S. Fan, X. Zhang, C. Yang, Spatiotemporal mode-locking in lasers with large modal dispersion. *Phys. Rev. Lett.* **126**, 093901 (2021).

41. K. Ji, I. Davidson, J. Sahu, D. J. Richardson, S. Wabnitz, M. Guasoni, Mode attraction, rejection and control in nonlinear multimode optics. *Nat. Commun.* **14**, 7704 (2023).

42. K. Krupa, A. Tonello, B. M. Shalaby, M. Fabert, A. Barthélémy, G. Millot, S. Wabnitz, V. Couderc, Spatial beam self-cleaning in multimode fibres. *Nat. Photonics* **11**, 237–241 (2017).

43. A. Dubietis, G. Tamošauskas, R. Šuminas, V. Jukna, A. Couairon, Ultrafast supercontinuum generation in bulk condensed media. *Lith. J. Phys.* **57**, 113–157 (2017).

44. C. Xu, W. W. Webb, *Topics in Fluorescence Spectroscopy: Nonlinear and Two-Photon-Induced Fluorescence* (Springer, 2002). vol. 5, pp. 471–540.

45. Y. Hontani, F. Xia, C. Xu, Multicolor three-photon fluorescence imaging with single-wavelength excitation deep in mouse brain. *Sci. Adv.* **7**, eabf3531 (2021).

46. S. Zhang, Z. Wan, R. D. Kamm, Vascularized organoids on a chip: Strategies for engineering organoids with functional vasculature. *Lab Chip* **21**, 473–488 (2021).

47. Z. Wan, A. X. Zhong, S. Zhang, G. Pavlou, M. F. Coughlin, S. E. Shelton, H. T. Nguyen, J. H. Lorch, D. A. Barbie, R. D. Kamm, A robust method for perfusable microvascular network formation in vitro. *Small Methods* **6**, 2200143 (2022).

48. D. G. Ouzounov, T. Wang, M. Wang, D. D. Feng, N. G. Horton, J. C. Cruz-Hernández, Y.-T. Cheng, J. Reimer, A. S. Tolias, N. Nishimura, C. Xu, In vivo three-photon imaging of activity of GCaMP6-labeled neurons deep in intact mouse brain. *Nat. Methods* **14**, 388–390 (2017).

49. C. Hajal, G. S. Offeddu, Y. Shin, S. Zhang, O. Morozova, D. Hickman, C. G. Knutson, R. D. Kamm, Engineered human blood-brain barrier microfluidic model for vascular permeability analyses. *Nat. Protoc.* **17**, 95–128 (2022).

50. J. Schindelin, I. Arganda-Carreras, E. Frise, V. Kaynig, M. Longair, T. Pietzsch, S. Preibisch, C. Rueden, S. Saalfeld, B. Schmid, J.-Y. Tinevez, D. J. White, V. Hartenstein, K. Eliceiri, P. Tomancak, A. Cardona, Fiji: An open-source platform for biological-image analysis. *Nat. Methods* **9**, 676–682 (2012).

51. I. Georgakoudi, K. P. Quinn, Optical imaging using endogenous contrast to assess metabolic state. *Annu. Rev. Biomed. Eng.* **14**, 351–367 (2012).

52. M. S. Pochechuev, A. A. Lanin, I. V. Kelmanson, D. S. Bilan, D. A. Kotova, A. S. Chebotarev, V. Tarabykin, A. B. Fedotov, V. V. Belousov, A. M. Zheltikov, Stain-free subcellular-resolution astrocyte imaging using third-harmonic generation. *Opt. Lett.* **44**, 3166–3169 (2019).

53. M. Quintero, S. L. Colombo, A. Godfrey, S. Moncada, Mitochondria as signaling organelles in the vascular endothelium. *Proc. Natl. Acad. Sci. U.S.A.* **103**, 5379–5384 (2006).

54. K. D. Falkenberg, K. Rohlenova, Y. Luo, P. Carmeliet, The metabolic engine of endothelial cells. *Nat. Metab.* **1**, 937–946 (2019).

55. C. M. Peppiatt, C. Howarth, P. Mobbs, D. Attwell, Bidirectional control of CNS capillary diameter by pericytes. *Nature* **443**, 700–704 (2006).

56. C. N. Hall, C. Reynell, B. Gesslein, N. B. Hamilton, A. Mishra, B. A. Sutherland, F. M. O'Farrell, A. M. Buchan, M. Lauritzen, D. Attwell, Capillary pericytes regulate cerebral blood flow in health and disease. *Nature* **508**, 55–60 (2014).

57. F. Fernández-Klett, N. Offenhauser, U. Dirnagl, J. Priller, U. Lindauer, Pericytes in capillaries are contractile *in vivo*, but arterioles mediate functional hyperemia in the mouse brain. *Proc. Natl. Acad. Sci. U.S.A.* **107**, 22290–22295 (2010).

58. S. Witte, A. Negrean, J. C. Lodder, C. P. De Kock, G. T. Silva, H. D. Mansvelder, M. L. Groot, Label-free live brain imaging and targeted patching with third-harmonic generation microscopy. *Proc. Natl. Acad. Sci. U.S.A.* **108**, 5970–5975 (2011).

59. S. Zhang, Z. Wan, G. Pavlou, A. X. Zhong, L. Xu, R. D. Kamm, Interstitial flow promotes the formation of functional microvascular networks in vitro through upregulation of matrix metalloproteinase-2. *Adv. Funct. Mater.* **32**, 2206767 (2022).

60. M. G. V. Heiden, L. C. Cantley, C. B. Thompson, Understanding the Warburg effect: The metabolic requirements of cell proliferation. *Science* **324**, 1029–1033 (2009).

61. J. A. Mosier, Y. Wu, C. A. Reinhart-King, Recent advances in understanding the role of metabolic heterogeneities in cell migration. *Fac. Rev.* **10**, 8 (2021).

62. M. R. Zanotelli, Z. E. Goldblatt, J. P. Miller, F. Bordeleau, J. Li, J. A. VanderBurgh, M. C. Lampi, M. R. King, C. A. Reinhart-King, Regulation of ATP utilization during metastatic cell migration by collagen architecture. *Mol. Biol. Cell.* **29**, 1–74 (2018).

63. D. Williams, B. Fingleton, Measurement of metabolites from migrating cells. *Metastasis* **2294**, 143–150 (2021).

64. D. Bhattacharya, A. P. Azambuja, M. Simoes-Costa, Metabolic reprogramming promotes neural crest migration via Yap/Tead signaling. *Dev. Cell* **53**, 199–211.e6 (2020).

65. H. Semba, N. Takeda, T. Isagawa, Y. Sugiura, K. Honda, M. Wake, H. Miyazawa, Y. Yamaguchi, M. Miura, D. M. Jenkins, H. Choi, J.-W. Kim, M. Asagiri, A. S. Cowburn, H. Abe, K. Soma, K. Koyama, M. Katoh, K. Sayama, N. Goda, R. S. Johnson, I. Manabe, R. Nagai, I. Komuro, HIF-1α-PDK1 axis-induced active glycolysis plays an essential role in macrophage migratory capacity. *Nat. Commun.* **7**, 11635 (2016).

66. M. A. Selak, S. M. Armour, E. D. MacKenzie, H. Boulahbel, D. G. Watson, K. D. Mansfield, Y. Pan, M. C. Simon, C. B. Thompson, E. Gottlieb, Succinate links TCA cycle dysfunction to oncogenesis by inhibiting HIF-α prolyl hydroxylase. *Cancer Cell* **7**, 77–85 (2005).

67. E. M. Palsson-McDermott, A. M. Curtis, G. Goel, M. A. Lauterbach, F. J. Sheedy, L. E. Gleeson, M. W. M. van den Bosch, S. R. Quinn, R. Domingo-Fernandez, D. G. W. Johnston, J.-K. Jiang, W. J. Israelsen, J. Keane, C. Thomas, C. Clish, M. V. Heiden, R. J. Xavier, L. A. J. O'Neill, Pyruvate kinase m2 regulates Hif-1α activity and IL-1β induction and is a critical determinant of the warburg effect in LPS-activated macrophages. *Cell Metab.* **21**, 65–80 (2015).

68. J. M. Gunn, B. Xu, J. M. D. Cruz, V. V. Lozovoy, M. Dantus. *Commercial and Biomedical Applications of Ultrafast Lasers VI* (SPIE, 2006), vol. 6108, pp. 61–68.

69. A. De la Cadena, J. Park, K. F. Tehrani, C. A. Renteria, G. L. Monroy, S. A. Boppart, Simultaneous label-free autofluorescence multi-harmonic microscopy driven by the supercontinuum generated from a bulk nonlinear crystal. *Biomed. Opt. Express* **15**, 491–505 (2024).

70. B. Li, C. Wu, M. Wang, K. Charan, C. Xu, An adaptive excitation source for high-speed multiphoton microscopy. *Nat. Methods* **17**, 163–166 (2020).

71. N. Ji, J. C. Magee, E. Betzig, High-speed, low-photodamage nonlinear imaging using passive pulse splitters. *Nat. Methods* **5**, 197–202 (2008).

72. K. Wang, W. Sun, C. T. Richie, B. K. Harvey, E. Betzig, N. Ji, Direct wavefront sensing for high-resolution in vivo imaging in scattering tissue. *Nat. Commun.* **6**, 7276 (2015).

73. A. Dvornikov, L. Malacrida, E. Gratton, The DIVER microscope for imaging in scattering media. *Methods Protoc.* **2**, 53 (2019).

74. I. Georgakoudi, K. P. Quinn, Label-free optical metabolic imaging in cells and tissues. *Annu. Rev. Biomed. Eng.* **25**, 413–443 (2023).

75. W. L. Rice, D. L. Kaplan, I. Georgakoudi, Two-photon microscopy for non-invasive, quantitative monitoring of stem cell differentiation. *PLOS ONE* **5**, e10075 (2010).

76. J. M. Levitt, M. E. McLaughlin-Drubin, K. Münger, I. Georgakoudi, Automated biochemical, morphological, and organizational assessment of precancerous changes from endogenous two-photon fluorescence images. *PLOS ONE* **6**, e24765 (2011).

77. A. Varone, J. Xylas, K. P. Quinn, D. Pouli, G. Sridharan, M. E. McLaughlin-Drubin, C. Alonzo, K. Lee, K. Münger, I. Georgakoudi, Endogenous two-photon fluorescence imaging elucidates metabolic changes related to enhanced glycolysis and glutamine consumption in precancerous epithelial tissues. *Cancer Res.* **74**, 3067–3075 (2014).

78. C. Stringer, T. Wang, M. Michaelos, M. Pachitariu, Cellpose: A generalist algorithm for cellular segmentation. *Nat. Methods* **18**, 100–106 (2021).

79. J. M. Riendeau, A. Gillette, E. Contreras Guzman, M. Costa Cruz, A. Kralovec, S. Udgata, A. Schmitz, D. A. Deming, B. A. Cimini, M. C. Skala, Cellpose as a reliable method for single-cell segmentation of autofluorescence microscopy images. bioRxiv 597994 [Preprint] (2024). https://doi.org/10.1101/2024.06.07.597994.

80. J. Pawley, *Handbook of biological confocal microscopy* (Springer Science & Business Media, 2006), vol. 236.

81. E. H. Stelzer, F. Strobl, B.-J. Chang, F. Preusser, S. Preibisch, K. McDole, R. Fiolka, Light sheet fluorescence microscopy. *Nat. Rev. Methods Primers* **1**, 73 (2021).

82. E. C. Ko, S. Spitz, F. M. Pramotton, O. M. Barr, C. Xu, G. Pavlou, S. Zhang, A. Tsai, A. Maaser-Hecker, M. Jorfi, S. H. Choi, R. E. Tanzi, R. D. Kamm, Accelerating the in vitro emulation of Alzheimer's disease-associated phenotypes using a novel 3D blood-brain barrier neurosphere co-culture model. *Front. Bioeng. Biotechnol.* **11**, 1251195 (2023).

83. C. Xu, W. W. Webb, Measurement of two-photon excitation cross sections of molecular fluorophores with data from 690 to 1050 nm. *J. Opt. Soc. Am. B* **13**, 481–491 (1996).

84. W. R. Zipfel, R. M. Williams, R. Christie, A. Y. Nikitin, B. T. Hyman, W. W. Webb, Live tissue intrinsic emission microscopy using multiphoton-excited native fluorescence and second harmonic generation. *Proc. Natl. Acad. Sci. U.S.A.* **100**, 7075–7080 (2003).

85. A. K. LaViolette, D. G. Ouzounov, C. Xu, Measurement of three-photon excitation cross-sections of fluorescein from 1154 nm to 1500 nm. *Biomed. Opt. Express* **14**, 4369–4382 (2023).

86. K. Charan, B. Li, M. Wang, C. P. Lin, C. Xu, Fiber-based tunable repetition rate source for deep tissue two-photon fluorescence microscopy. *Biomed. Opt. Express* **9**, 2304–2311 (2018).

87. G. Donnert, C. Eggeling, S. W. Hell, Major signal increase in fluorescence microscopy through dark-state relaxation. *Nat. Methods* **4**, 81–86 (2007).

88. N. Vora, C. M. Polleys, F. Sakellariou, G. Georgalis, H.-T. Thieu, E. M. Genega, N. Jahanseir, A. Patra, E. Miller, I. Georgakoudi, Restoration of metabolic functional metrics from label-free, two-photon human tissue images using multiscale deep-learning-based denoising algorithms. *J. Biomed. Opt.* **28**, 126006–126006 (2023).
